# Regulation of Sirtuin-Mediated Protein Deacetylation by Cardioprotective Phytochemicals

**DOI:** 10.1155/2017/1750306

**Published:** 2017-11-06

**Authors:** Niria Treviño-Saldaña, Gerardo García-Rivas

**Affiliations:** ^1^Cátedra de Cardiología y Medicina Vascular, Escuela de Medicina y Ciencias de la Salud, Tecnológico de Monterrey, Monterrey, Mexico; ^2^Centro de Investigación Biomédica, Hospital Zambrano Hellion, Tec Salud del Sistema, Tecnológico de Monterrey, Monterrey, Mexico

## Abstract

Modulation of posttranslational modifications (PTMs), such as protein acetylation, is considered a novel therapeutic strategy to combat the development and progression of cardiovascular diseases. Protein hyperacetylation is associated with the development of numerous cardiovascular diseases, including atherosclerosis, hypertension, cardiac hypertrophy, and heart failure. In addition, decreased expression and activity of the deacetylases Sirt1, Sirt3, and Sirt6 have been linked to the development and progression of cardiac dysfunction. Several phytochemicals exert cardioprotective effects by regulating protein acetylation levels. These effects are mainly exerted via activation of Sirt1 and Sirt3 and inhibition of acetyltransferases. Numerous studies support a cardioprotective role for sirtuin activators (e.g., resveratrol), as well as other emerging modulators of protein acetylation, including curcumin, honokiol, oroxilyn A, quercetin, epigallocatechin-3-gallate, bakuchiol, tyrosol, and berberine. Studies also point to a cardioprotective role for various nonaromatic molecules, such as docosahexaenoic acid, alpha-lipoic acid, sulforaphane, and caffeic acid ethanolamide. Here, we review the vast evidence from the bench to the clinical setting for the potential cardioprotective roles of various phytochemicals in the modulation of sirtuin-mediated deacetylation.

## 1. Introduction

Cardiovascular diseases (CVDs) have remained the leading cause of death worldwide for the past two decades. In 2012, coronary artery disease alone was responsible for 7.4 million deaths, and CVDs accounted for 17.3 million deaths worldwide [1]. Based on current estimates, the number of CVD-related deaths is expected to increase to 23.6 million by 2030 [1]. Coronary heart disease, hypertension, peripheral vascular disease, myocardial infarctions, strokes, and heart failure are the most prevalent CVDs [2]. Their aetiology is diverse and includes various risk factors, such as aging, obesity, smoking, and diabetes [1]. Abundant natural biologically active substances, also known as functional molecules, from diverse sources can potentially decrease risk factors for CVDs, thereby significantly reducing the incidence of CVDs [3–7].

The search for new functional molecules to prevent CVDs faces a major challenge due to the growing list of pathogenic mechanisms attributed to a multitude of interrelated pathways. Research on the role of posttranslational modifications (PTMs) in the development and progression of CVDs has spiked dramatically, with modulation of PTMs currently considered a potential therapeutic strategy [8–10].

PTMs are important regulators of the synthesis, subcellular localization, and enzymatic activity of proteins. PTMs also modulate signal transduction pathways and cellular metabolism (reviewed in [11]). PTMs respond rapidly to both internal and external (environmental) stimulation, allowing for efficient signal transmission and amplification. Among PTMs, most research thus far has focused on protein phosphorylation, although protein acetylation has emerged as a key regulatory mechanism and an attractive therapeutic target in the field of chronic diseases ([8–10], reviewed in [12]). Although acetylation was first described in the early 1960s, detailed studies of its biological role did not take place until the late 1990s. The first description of protein acetylation focused on histones as targets of histone acetyltransferases, which transfer acetyl groups from acetyl coenzyme A to specific lysine residues on histones, exposing DNA to transcription [13]. Conversely, histone deacetylases (HDACs) remove acetyl groups from acetylated lysine amino acids on histones, allowing chromatin condensation and therefore silencing of gene transcription [13]. Later research described the deacetylation of histone and nonhistone proteins, orchestrated by a subgroup of deacetylases known as sirtuins [14]. A classification system of HDACs was later developed based on their molecular targets and mechanism of action. Briefly, there are four classes of HDACs in mammals, all of which utilize zinc as a cofactor, except for sirtuins (class III), which utilize nicotinamide dinucleotide (NAD^+^) as a substrate in enzymatic activity [14, 15]. As NAD^+^ levels vary according to nutrient availability, the activity of sirtuins is strongly associated with the metabolic status of the cell, with their activity increasing under conditions of calorie restriction and exercise [15–17].

**Figure 1:**
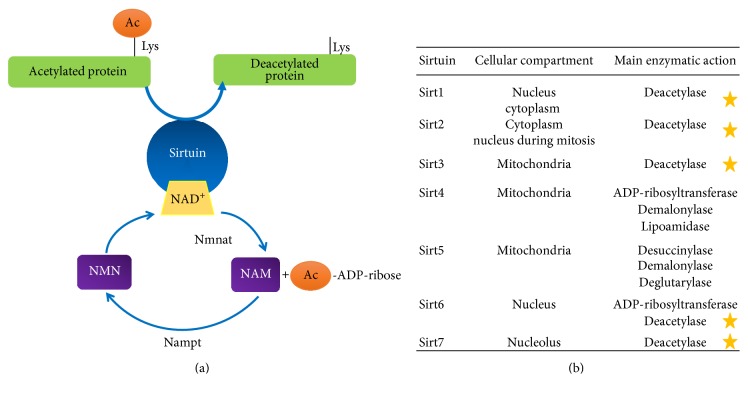
(a) NAD^+^-dependent deacetylation reaction performed by sirtuins. NAD^+^ is synthesized from its precursor NMN and degraded into NAM + acetyl-ADP-ribose once sirtuins utilize it for their activation [10–12]. Activated sirtuins interact with their target protein and transfer the acetyl group from target lysine residues to ADP-ribose. (b) Sirtuins1–7, their subcellular localization, and the enzymatic activity they perform; yellow stars indicate deacetylase activity [13, 14, 17, 18]. NAD: nicotinamide dinucleotide; NMN: nicotinamide mononucleotide; Nmnat: nicotinamide mononucleotide adenylyltransferase; Nampt: nicotinamide phosphoribosyltransferase.

Sirtuins are widely distributed in prokaryotic and eukaryotic species, with seven isoforms characterized in humans [18, 19]. They share a common catalytic domain but differ with regard to their N- and C-terminal sequences, which determine their susceptibility to regulation by sirtuin-activating compounds [20] and PTMs (reviewed in [21]). The molecular targets and subcellular localization of different sirtuins vary, with some sirtuins present in more than one organelle. Although all sirtuins are classified as deacetylases, Sirt4 and Sirt5 have weak deacetylase activity and function as demalonylases and deacylases [22, 23] (Figure 1).

## 2. Protein Acetylation and CVDs

**Figure 2:**
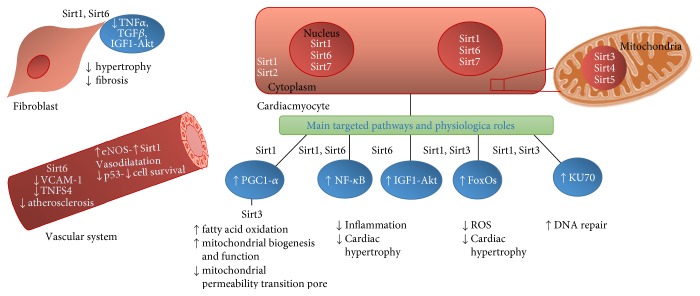
Targeted pathways by sirtuins in cardiac fibroblasts, cardiac myocytes, and in the vascular system. Sirt1 and Sirt6 prevent fibrosis and fibroblast hypertrophy by repressing growth factors such as TGF-*β* and IGF1, as well as inflammatory cytokines like TNF-*α* [24, 25]. At the vascular level, Sirt1 activation induces vasodilatation and promotes cell survival via deacetylation of eNOS and p53. The activity of eNOS and p53 increases in a Sirt1-dependent manner [26], whereas Sirt6 inhibits VCAM and TNFS protecting against atherosclerosis [27]. Sirt1 in the cardiac myocyte promotes mitochondrial biogenesis and function mainly through the activation of PGC1-*α* and Sirt3 [28], which activates mitochondrial dehydrogenases, enzymes from the electron transport chain, and the synthase and represses cyclophilin D, protecting the cell from the opening of the mitochondrial permeability transition pore [29–38]. Nuclear sirtuins 1 and 6 prevent cardiac hypertrophy and inflammation through the inactivation of the NF-*κ*B pathway [24, 25], as well as IGF-Akt by Sirtuin 6 [25]. Sirtuins 1 and 3 are also regulators of oxidative stress through the regulation of FoxOs, and both promote DNA repair through the activation of Ku70 [39–42].

Research on the impact of protein acetylation in CVDs has focused mainly on Sirt1, Sirt3, and Sirt6, with little information regarding the function of the remaining sirtuins. Sirt1 and Sirt3 are involved in the regulation of important cellular mechanisms (e.g., apoptosis and cell survival), as well as the regulation of reactive oxygen species levels, hypertrophic and fibrotic responses, and mitochondrial biogenesis and function (Figure 2). Recent evidence suggests that impairments in the sirtuin family are associated with the development and progression of CVDs, as discussed below. Sirt1 modulates early embryogenesis, and homozygous *sirt1^−/−^* mice die shortly after birth [43]. As shown in deletion studies of crossbred mice, *sirt1^−/+^* mice develop dilated cardiomyopathy [44] and exhibit increased cardiac injury induced by ischemia/reperfusion (I/R) [45]. These deleterious effects are associated with inhibition of Sirt1-mediated activation of forkhead box protein O1 (FOXO1) and O3 (FOXO3), which are responsible for the transcription of antioxidant enzymes, such as superoxide dismutase and catalases [44–46]. Sirt1 deficiency results in activation of proliferative and proinflammatory pathways involving tumour necrosis factor-*α* and nuclear factor-*κ*B (NF-*κ*B), leading to cardiac hypertrophy, fibrosis, and heart failure [24, 45] (Figure 2). With aging, the deleterious effects of Sirt1 deficiency become more prominent [47, 48]. Sirt1 stimulation and overexpression appears to provide protection against age-related cardiac diseases [48–50]. In addition, low to moderate overexpression of Sirt1 (2.5–7.5 times) mitigates cardiac hypertrophy induced by aging [48], provides protection against oxidative stress-induced cardiotoxicity [45], and improves endothelial function [26, 51]. Sirt1 stimulation and overexpression also has a range of other beneficial effects (Table 1). Sirt1 expression increases (12-fold) in hearts of dogs with experimental heart failure induced by rapid pacing [55]. Transgenic mice with cardiac-specific overexpression (20-fold) of Sirt1 exhibit mitochondrial dysfunction and dilated cardiomyopathy [64]. Thus, it appears that moderate stimulation of Sirt1 is beneficial for cardiac function, whereas excessive stimulation has deleterious effects on the heart.

Sirt3 regulates mitochondrial protein acetylation, and severe hyperacetylation of mitochondrial proteins occurs in *sirt3^−/−^* knockout mice [29]. Such hyperacetylation has direct implications for ATP-dependent cellular processes, as hyperacetylation of enzymes involved in the Krebs cycle and electron transport chain translates into severe depletion of ATP (as much as 50%) [30], as well as compromised cardiac myocyte function. As demonstrated in previous studies, *sirt3^−/−^* mice develop cardiac hypertrophy, fibrosis, and mitochondrial dysfunction in an age-dependent manner [31]. In addition, *sirt3^−/−^* mice are more sensitive to damage induced by I/R injury [32–35] and microvascular dysfunction [34]. In contrast, overexpression of Sirt3 in mice hearts provides protection against cardiac hypertrophy and fibrosis [36, 37]. It also provides protection against oxidative stress-induced damage and apoptosis in the myocardium [38]. These protective effects are associated with activation of the antioxidant defence response, mediated by FoxO3a, which preserves mitochondrial energy production via the activation of mitochondrial dehydrogenases, thereby preventing mitochondrial permeability transition pore (mPTP) opening [38]. mPTP opening is followed by increases in Ca^2+^ overload, in addition to depletion of ATP and mitochondrial swelling, which eventually cause necrosis and apoptosis in cardiac myocytes [65]. In animal models of metabolic syndrome and ventricular dysfunction, mitochondria are prone to mPTP opening as compared with controls, concomitant with decreased Sirt3 expression and a hyperacetylated mitochondrial profile [66]. In biopsies of patients with heart failure, Sirt3 expression was lower in obese patients than in nonobese patients. Interestingly, acetylation profiles of patients with end-stage heart failure are correlated with body mass index and cardiac remodelling [66].

As shown in similar studies, the level of protein acetylation is closely associated with the metabolic status of the cell, and it varies with nutritional status [33]. Moreover, persistent hyperacetylation in the heart, as occurs in Sirt3 knockout mice, results in increased sensitivity to hemodynamic stress [67]. In addition, an increase in the NADH : NAD ratio inhibits Sirt3, resulting in mitochondrial hyperacetylation [67]. Restoring Sirt3 activity via normalization of the NADH/NAD ratio reverses protein hyperacetylation in complex I-deficient hearts, as well as in hearts with cardiac remodelling. Thousands of mitochondrial acetylation sites have been identified in acetylome analyses of *sirt3*^*−*/−^ mice [68] and human failing hearts [8]. Among these, hyperacetylation of the malate-aspartate shuttle and regulators of mPTP opening are linked to the development of cardiac dysfunction [68]. Significant mitochondrial lysine hyperacetylation occurs in humans with end-stage heart failure, as shown by a myocardial acetylproteomic study [8].

Of note, homozygous *sirt3*^*−*/−^ mice do not express any specific phenotype at birth, with cardiac development and function appearing normal under physiological conditions. Nevertheless, when exposed to I/R injury or agonist-induced cardiac hypertrophy, *sirt3*^*−*/−^ mice exhibit severe mitochondrial hyperacetylation, in addition to decreased mitochondrial and myocardial function and lower survival rates [30]. This highlights the role of Sirt3 in the maladaptation observed during stressful cardiac conditions [35] and suggests that an NAD precursor, as well as sirtuin-activating compounds (STACs), could be used as cardiac therapy.

Sirt6 is a negative regulator of the insulin-like growth factor-1-protein kinase B pathway, which is implicated in the development of heart failure. Although *sirt6^−/−^* mice develop cardiac hypertrophy and heart failure, transgenic mice overexpressing this sirtuin are protected against both events. Likewise, Sirt6 expression levels are reduced in patients with failing hearts and in those with atherosclerosis [25, 69]. Sirt6 expression levels are also decreased in murine models [25]. Notably, *sirt6^−/−^* deficiency is associated with overexpression of proinflammatory cytokines, such as tumour necrosis factor superfamily member 4 and vascular cell adhesion molecule 1. These findings suggest that maintaining Sirt6 expression might be a novel therapeutic strategy against both cardiac and vascular dysfunction. Figure 2 summarizes the main reported targets and physiological roles of sirtuins in CVD prevention.

## 3. Regulation by Phenolic Compounds and Synthetic Molecules of Protein Acetylation in CVDs

**Figure 3:**
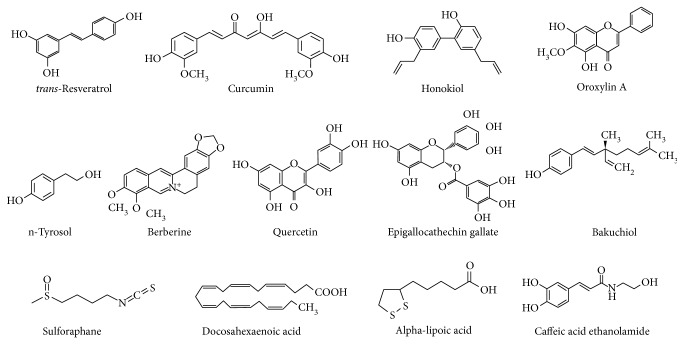
Phytochemicals with beneficial effects in CVDs through modulation of protein acetylation.

The potential roles of several molecules as sirtuin activators have been studied due to their cardioprotective effects, which have been described both *in vitro* and *in vivo* (Figure 3). Most of these molecules are phenolic compounds, such as resveratrol (trans-3,5,4′-trihydroxystilbene) [17], but some synthetic molecules have been developed and successfully tested [20]. As phenolic compounds are hydrophobic, they can permeate the cell membrane to perform biological functions. The rate at which they enter the cell depends on both the size of the molecule and hydrophobicity of the attached functional groups [27, 70].

The mechanism by which phenolic molecules promote the activity of sirtuins, specifically that of Sirt1, may involve allosteric activation and direct binding to a negatively charged amino acid from the N terminus, [54]. The binding of STACs lowers the Km of the substrate and thus enhances enzymatic activity. Although sirtuins share a common catalytic domain, they differ in their N- and C-terminal sequences. Thus, the mechanism by which phenolic molecules promote the activity of Sirt1 cannot be generalized to other sirtuin isoforms. As shown in previous research, activators of other sirtuins, such as Sirt3, directly interact with the protein, but the specific mechanism remains unclear [37]. Targeting adenosine monophosphate protein kinases (AMPKs) upregulates both Sirt1 activity and that of the peroxisome proliferator-activated receptor gamma coactivator 1-*α* (PGC1-*α*), a transcriptional coactivator, thereby indirectly increasing nuclear and mitochondrial sirtuin activity [28]. Other approaches for Sirt activation involve NAD^+^ precursors, such as nicotinamide riboside or nicotinamide mononucleotide [71], and augmentation of NAD^+^ availability via inhibition of glycohydrolases CD38 and CD157, which convert NAD^+^ to nicotinamide mononucleotide [72]. Detailed descriptions of the mechanism underlying the actions of STACs and the role of sirtuins in CVDs can be found elsewhere [73, 74]. The following sections provide a comprehensive review of natural STACs that regulate protein acetylation in CVDs.

### 3.1. Resveratrol

Resveratrol is a polyphenol found in grapes and red wine. It is one of the best studied phytochemicals, and it is known to provide protection against CVDs. It was first described as a Sirt1 activator by Howitz et al., who demonstrated that resveratrol was capable of reducing the Km of both the acetylated target of Sirt1 and that of NAD^+^ (35- and 5-fold, resp.) [17]. The mechanism underlying the activity of resveratrol was later questioned, with some proposing that it was dependent on the fluorophore utilized to label the evaluated peptide [75]. However, later research confirmed that resveratrol was an allosteric activator of Sirt1 [54].

Resveratrol has been evaluated in different models of cardiac disease, including chronic conditions such as heart failure and atherosclerosis, or damage associated with acute events, such as I/R (Table 1). *In vitro*, resveratrol decreases oxidative stress, inhibits hypertrophy, promotes cell survival, and inhibits apoptosis [38, 52, 56, 59, 60, 62, 76]. *In vivo*, supplementation with resveratrol decreases hypertrophy and fibrosis [52]. It also preserves cardiac function in models of heart failure induced by norepinephrine [60], doxorubicin [56–58], steptozotocin [61], and angiotensin II [53]. Furthermore, resveratrol prevents cardiac dysfunction in models of acute myocardial infarction induced by left coronary flow occlusion [76]. These cardioprotective effects are dependent on the activation of Sirt1 [52, 56, 59, 60, 62, 76] and Sirt3 [38, 53], as both gene silencing and specific sirtuin antagonists block the beneficial response. Moreover, in models of atherosclerosis, such as the apolipoprotein E (*apoE*^−/−^) mouse, resveratrol reverses endothelial nitric oxide synthase (eNOS) uncoupling and reduces oxidative stress. As resveratrol also enhances the activity of FoxO, the underlying mechanism possibly involves FoxO via NAD-dependent deacetylation, which contributes to cellular stress resistance [39]. Although sirtuin expression has not been studied in this model, treatment with sirtinol, a sirtuin inhibitor, abolished the beneficial effects of resveratrol supplementation [63].

The decrease in the activity of sirtuins with age is well known. Therefore, several models have studied the effects of supplementation with resveratrol on cardiac disease in a senescence setting. In senescence-accelerated mice, supplementation with resveratrol resulted in the recovery of Sirt1 activity [40]. Resveratrol supplementation also provided protection against hypertrophy and apoptosis, as well as preservation of left ventricular function, as compared with hearts from unsupplemented aged mice [40]. The addition of resveratrol to an exercise regime in aged rats potentiated the increase in Sirt1 activity achieved by exercise alone, and this translated into decreased fibrosis, apoptosis, and improved fractional shortening [41]. The molecular pathways involved in these *in vitro* and *in vivo* studies are summarized in Table 1.

### 3.2. Curcumin

Curcumin (diferuloylmethane), a polyphenol derived from the turmeric plant, is the second most well-studied phenolic compound for the treatment of CVDs. It modulates cardiac acetylation, mainly via the stimulation of Sirt1 [42, 77–79] and inhibition of histone acetyltransferase p-300 (p-300-HAT) [80–84].

The potential of curcumin-induced activation of Sirt1 as a mechanism to improve vascular function was studied in human THP-1-machrophage-derived foam cells [77]. Curcumin activated Sirt1 and decreased cellular cholesterol levels, preventing the formation of atherosclerotic plaques [77]. The authors attributed their findings to Sirt1-dependent activation of the ATP binding transporter cassette 1, which increased cholesterol efflux [77]. Another study showed that curcumin improved vascular function by Sirt1-dependent activation of eNOS [79]. By deacetylating eNOS, Sirt1 stimulated endothelium-dependent NO synthesis and protected endothelial cells against premature senescence induced by oxidative stress [79].

Curcumin-induced inhibition of p300-HAT is associated with decreased acetylation, which provides protection against cardiac injury. In murine models of myocardial infraction, curcumin-induced inhibition of p300-HAT resulted in decreased infract sizes, in addition to the prevention of cardiac hypertrophy and fibrosis and preservation of ventricular function [42, 80, 81]. The beneficial effects of curcumin have been attributed to is downregulation of transcription factors, such as NF-*κ*B, GATA binding protein 4, and transforming growth factor *β*1, that are normally activated in the presence of myocardial damage (Table 2). In models of both chronic and acute myocardial damage, curcumin-induced inhibition of p-300-HAT result in decreased apoptosis in response to deacetylation of p53, as well as inhibition of proapoptotic Bax and caspase 3 [42, 80]. These cardioprotective effects were attributed to Sirt1 stimulation [77, 79]. Based on the current literature, decreased cardiac acetylation, either by activation of Sirt1 or inhibition of p-300-HAT, appears to induce similar responses, such as inhibition of proinflammatory and profibrotic transcription factors, as well as activation of antioxidant enzymes, that ultimately preserve cardiac function (Table 2) [81–83].

### 3.3. Honokiol

Honokiol, a biphenolic compound obtained from the bark of the magnolia tree, was recently evaluated in a murine model of cardiac hypertrophy and fibrosis [37]. Remarkably, the authors demonstrated that honokiol was not only capable of preventing agonist-induced heart failure but it also reversed preexisting fibrosis and ventricular failure. The cardioprotective effects of honokiol were associated with a dose-dependent increase in Sirt3 activity. Regarding the mechanism of action, the authors showed that honokiol entered mitochondria and directly interacted with Sirt3, although the precise binding site for activation remains unclear.

### 3.4. Oroxylin A

Oroxylin A (OA) is derived from the root of *Scutellaria baicalensis.* Based on its chemical structure, with hydroxyl groups at C-5 and C-7 and a methoxy group at C-6, it is classified as a flavone [85]. As demonstrated in pharmacokinetic studies involving animal models, OA is highly bioavailable after oral infection, which increases its potential as a bioactive compound [85]. Previous studies reported that OA functioned as a Sirt3 activator in human breast cancer cells [86] and as an acute Sirt3 activator in an *in vitro* model of cardiac myocyte insulin resistance [87, 88]. Via the activation of Sirt3, OA prevented loss of contractile function in response to insulin overstimulation, as evidenced by preserved peak shortening [88]. OA also appeared to reduce angiotensin-induced hypertrophy and cell death in cardiac myoblasts, pointing to a potential cardioprotective effect. In addition, OA decreased mitochondrial hyperacetylation and energetic debacle in a dose-dependent manner [89]. Based on the current evidence, OA appears to increase Sirt3 activity in cardiac cells, although no precise mechanism of action has been described thus far.

### 3.5. Other Emerging Regulators of Protein Acetylation in CVDs

Information on regulators of protein acetylation other than the aforementioned is scarce but encouraging. Details on phytochemicals capable of modulating cardiac acetylation via the activation of sirtuins in models of CVDs are presented in Figure 3. They include quercetin, epigallocatechin-3-gallate, bakuchiol, tyrosol, and berberine [81–86]. Nutraceuticals that function as activators of Sirt1 include docosahexaenoic acid, alpha-lipoic acid, sulforaphane, and caffeic acid ethanolamide, although the mechanisms by which they activate sirtuins remain to be elucidated [26, 89–97]. Most of these functional molecules share common molecular targets and exert their actions, for example, by stimulation of AMPK-*α*, eNOS, PGC1-*α*, and superoxide dismutase or inhibition of NF-*κ*B and proapoptotic molecules (e.g., Bax and caspase 3).  Table 3 summarizes the findings of experimental models and the results obtained for each reported phytochemical, specifying the molecular pathways and targets involved.

## 4. Clinical Evidence for Cardioprotective Properties of Natural Modulators of Protein Acetylation

Experimental data supports cardioprotective properties of natural modulators of protein acetylation, but little is known about their effects in a clinical setting. To date, resveratrol is the only phytochemical that has been tested as a sirtuin activator in humans. As discussed previously, resveratrol mimics calorie restriction effects *in vitro* and *in vivo.* A recent study of obese patients demonstrated that supplementation with resveratrol for 30 days significantly increased Sirt1 expression via activation of AMPK and that it improved muscle mitochondrial respiration by increasing fatty acid oxidation [98]. This translated into decreased hepatic lipid accumulation and reduced inflammation [98]. The study did not measure variations in cardiac function. Nevertheless, it is well known that obesity is an independent risk factor for CVDs [1]. In this context, the observed protection against inflammation and lipid accumulation might decrease the risk of vascular and cardiac pathologies in obese patients.

In contrast to the synergetic effects of resveratrol and physical activity observed in murine models, supplementation with resveratrol blunted the cardioprotective effects achieved by 8 weeks of physical exercise in men over 60. This study detected no changes in Sirt1 expression in either group [99]. As the roles of both physical activity and resveratrol as activators of Sirt1 have been demonstrated in experimental models of senescence, it appears that the human response to both stimuli is different with regard to the activation of the AMPK/Sirt1/PGC1-*α* axis, as discussed previously [100].

The role of resveratrol as a Sirt1 activator was evaluated in postmenopausal woman of with a normal weight and glucose tolerance [101]. In this group, resveratrol supplementation was associated with no major improvements in metabolic parameters or modification of Sirt1 expression [101]. Although sirtuin deficiency is uncommon in healthy individuals, it has been reported in obese patients and patients with metabolic syndrome [98, 102]. Additionally, sirtuin activity was decreased in a study of heart failure patients [8]. In common with vitamin supplementation in the absence of any vitamin deficit [103], supplementation with sirtuin activators is not expected to have any benefit when basal levels of Sirt1 expression and activity are normal. The aforementioned might explain why supplementation with resveratrol has a positive effect under conditions of obesity [98] but no effects under conditions of normal weight and glucose tolerance [101].

## 5. Bioavailability of Cardioprotective Phytochemicals

Although experimental models have revealed promising cardioprotective effects of phytochemicals, their bioactivity in the clinical setting remains to be explored. After oral ingestion of phenolic compounds, two factors mainly determine their biological activity: absorption and metabolic stability. Absorption in the small intestine varies according to the hydrophobicity of the compounds and affinity of membrane transporters, and only aglycones can be efficiently absorbed [27, 104–106]. Most polyphenols must be hydrolysed by intestinal enzymes or microflora in order to permeate the intestinal epithelium [105, 106]. Once they are absorbed and reach the liver, their stability depends on their sensitivity to metabolism by phase II enzymes.

Only a few pharmacokinetic studies of phenolic compounds have been reported in humans. A study of resveratrol absorption and metabolism in healthy subjects showed that after oral ingestion of resveratrol, around 92% of the administered dose was excreted in urine and faeces [27]. In an experimental study, the final amount of resveratrol absorbed in liver fractions was extremely low due to rapid formation of conjugates of resveratrol, mainly by sulfation and glucuronidation [104].

Some studies have explored the potential impact of functional groups on the bioavailability of polyphenols. Methylated polyphenols easily permeated the intestinal epithelium, without previous conjugation, in contrast to unmethylated compounds [104]. In the presence of hepatic phase II enzymes, methylated polyphenols were more stable than their unmethylated counterparts. These properties should encourage more in-depth studies of the potential biological effects of naturally methylated polyphenols, considering the benefits of their pharmacokinetic profiles.

Recently, the role of nanocarriers as a novel strategy to increase the bioavailability of polyphenols has been studied (reviewed in [107]). Besides increasing absorption and protecting polyphenols from enzymatic degradation, nanocarriers can be configured to release material in a controlled and prolonged manner, maintaining bioactivity for longer periods. Two studies that examined the potential of nanocurcumin as a sirtuin activator reported significant improvements in its bioactivity [82, 84]. Although neither study directly compared the biological effects of curcumin versus those of nanocurcumin, the biological effects of nanocurcumin were observed at lower concentration than that of free curcumin [42, 80, 81] (Table 2).

A number of studies have examined other nanoencapsulated polyphenols, although they did not evaluate their activity as modulators of acetylation. Recent evidence indicates that cardiac muscle and vessels are targets for nanotechnology-based therapies that could reach the myocardium through dysfunctional permeable endothelium [108]. In a murine model of heart failure, passive cardiac accumulation of high concentrations of nanocarriers occurred after a single application [108]. Compared to normal heart tissues, the accumulation of nanovectors was more than 10 times higher in the heart failure murine model [108]. This approach using nanocarriers, represents a potential avenue for functional molecules, which may be translated into innovative treatments to improve patient CVD outcomes.

Recent studies revealed that the microbiome can significantly modify the extent to which phenolic compounds are metabolized [109, 110]. It is well known that intestinal and colonic microorganisms vary according to a patient's physiological status [111]. Thus, preliminary pharmacokinetic studies should ideally be performed in systems simulating both healthy and unhealthy gastrointestinal tracts.

## 6. Potential Toxicity of Phytochemicals

Although numerous health benefits are associated with the consumption of phytochemicals, caution is needed when selecting an exploratory dose because of their hormetic behaviour. Plants synthesize phytochemicals and activate adaptive molecular pathways to protect themselves against cellular stress. Although exogenous administration of phytochemicals to organisms can have protective effects at specific doses, they can also have prooxidant and cytotoxic effects at relatively high concentrations [112].

Regarding cardioprotection and the toxicity of phytochemicals, a recent study compared heart function in rats after 21 days of supplementation with increasing doses of resveratrol [113]. The authors reported that 2.5 mg/kg/d and 25 mg/kg/d protected ex vivo against I/R induced injury but that higher doses had adverse effects on cardiac function [113]. Interestingly, in this study, both rats and rabbits showed greater tolerance to a synthetic resveratrol formulation, Longevinex, which contains small amounts of quercetin and ferulic acid, than to resveratrol alone. This finding might be explained by increased flavonoid stability and metabolic competition when administered in combination than when administered singly, with the combination therapy potentially decreasing, as well as having a prooxidant effect. Clinical trials reported no adverse effects of resveratrol doses ranging from 0.4 mg/kg/d to 5 g/d [114, 115]. The apparent higher tolerance observed in humans than in animal models might be explained by lower resveratrol bioavailability and metabolic competition with other nutrients present in a patient's diet, in addition to differences in the gastrointestinal tracts of humans and animals.

To our knowledge, there are no reports on the pharmacokinetic profiles of the remaining reviewed molecules in humans. However, as shown by *in vitro* and *in vivo* studies, most phytochemicals exhibit a similar bimodal dose-response curve. Supplementation with epigallocatechin-3-gallate at 30 and 60 mg/kg abolished anxiety in mice [116, 117]. Remarkably, increasing the dose to 100 mg/kg induced 100% mortality in less than 24 h [116, 117]. Epigallocatechin-3-gallate is the most abundant catechin in green tea. Although moderate consumption of this beverage has been associated with health benefits, more than 1 L per day increased the risk of cancer in humans [118], although this finding was attributed to the temperature of the beverage and not only to its bioactive substances [118, 119]. Although many studies in the literature support the potential cardioprotective effects of the phytochemicals reviewed in the present work, in-depth studies of their bioavailability and pharmacokinetic profiles are missing. Further research is needed to address this issue.

## 7. Conclusion

Protein hyperacetylation is associated with the development of several CVDs, including atherosclerosis, hypertension, cardiac hypertrophy, and heart failure. The underlying mechanisms include activation of proinflammatory cytokines and proapoptotic molecules and inhibition of mitochondrial biogenesis and function, in addition to downregulation of enzymes involved in antioxidant defence. Decreased expression and activity of the deacetylases Sirt1, Sirt3, and Sirt6 are associated with the development and progression of the aforementioned pathologies.

**Figure 4:**
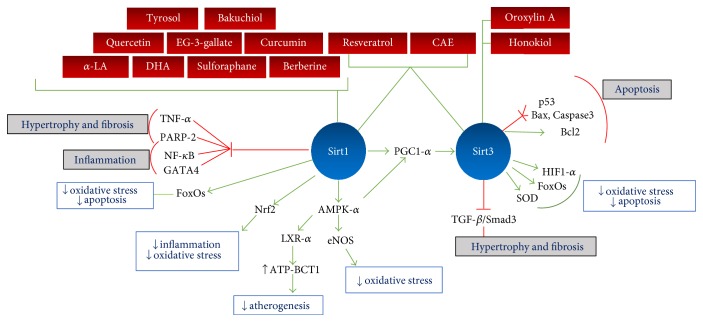
Cardioprotective effects of sirtuin activators and the molecular pathways involved. Red boxes state the phytochemicals regulating the activity of Sirt1, Sirt3, or both, as indicated by the green connecting lines. Green lines indicate activation of the indicated targets, whereas red lines indicate inhibition. Gray boxes indicate inhibition of cellular responses and white boxes indicate stimulation of them. CAE: caffeic acid ethanolamide; a-LA: alpha-lipoic acid; DHA docosahexaenoic acid.

The potential cardioprotective roles of several phytochemicals as regulators of sirtuin-mediated protein deacetylation have been studied. Preclinical evidence suggests that by activating Sirt1 and/or Sirt3, some bioactive phytochemicals can protect the cardiovascular system from the negative consequences of hyperacetylation (Figure 4). In the clinical setting, only resveratrol has been validated as a Sirt1 activator in obese patients, with conflicting results found in other clinical trials performed with men over 60 and postmenopausal women. As healthy subjects show no benefit from supplementation, it appears that sirtuin activators should be evaluated only in specific patient groups, such as obese subjects or those with metabolic syndrome or heart failure, with a previous reported deficiency. Pharmacokinetic studies in humans are required to determine the optimum dose selection. The low bioavailability of phytochemicals limits their biological effects. Various strategies, including nanodelivery systems, aimed at overcoming this problem are currently under way. Initial results of these studies appear promising.

## Figures and Tables

**Table 1:** Cardioprotective effect and mechanism of action of resveratrol in preclinical studies.

Target HDAC or HAT	Molecular pathway	Experimental model	Cardiovascular effect	Reference
↑ Sirt1	↑ PGC-1*α* ↑ Bcl2 ↓ Bax, caspase 3 ↑ SOD, SDH, Cyt-c oxidase	TAC induced myocardial infraction *In vivo* Hypoxia induced dysfunction *In vitro*	↑ LVEF ↓ fibrosis ↓ apoptosis	[52]
↑ Sirt3	↓ TGF-*β*/Smad3	TAC induced heart failure *In vivo*	↓ fibrosis ↓ collagen deposition ↓ cardiac hypertrophy Prevented decrease in cardiac FS Preserved diastolic function	[53]
↑ Sirt1	↑ SOD	Chronic heart failure model *In vivo* Ang II or antimycin A induced oxidative stress *In vitro*	↑ FS ↑ LVEF ↑ survival ↓ apoptosis	[54]
↑ Sirt3	↑ SOD	Dox-induced mitochondrial dysfunction *In vivo* *In vitro*	↓ oxidative stress ↑ ATP mitochondrial production	[32]
↑ Sirt1	↓ p38MAPK ↓ caspase 3 ↓ Bax ↑ Bcl-2 ↑ SOD1	Dox-induced heart failure *In vivo*	↑ FS ↓ apoptosis ↓ oxidative stress	[55]
↑ Sirt1	↑ AMPK	Dox-induced cardiotoxicity *In vitro*	↑ survival	[56]
↑ Sirt3	↓ p53 ↓ Bax, Cyt-c	Dox-induced cardiotoxicity *In vivo*	↓ apoptosis Attenuated loss of diastolic and systolic function.	[57]
↑ Sirt1	↓ USP7 ↓ p300 ↓ Bax, caspase 3 ↓ p53	Dox-induced cardiotoxicity in young and aged hearts *In vivo*	↑ FS ↑ EF ↓ LVEDS ↓ apoptosis	[58]
↑ Sirt1	↑ PI3K-Akt ↓ TNF-*α* ↓ FAS/FADD/caspase 8 ↓ caspase 3 ↑ FoxO3	Exercise during aging *In vivo*	↑ FS ↓ fibrosis ↓ apoptosis	[41]
↑ Sirt1	↓ ac-FoxO1 ↓ Bim, Bax ↓ p53	Aging *In vivo*	↑ FS ↑ LVEF ↓ fibrosis ↓ apoptosis	[40]
↑ Sirt1	↑ SOD ↑ GSH	High glucose-induced mitochondrial oxidative stress. *In vitro*	↓ oxidative stress	[59]
↑ Sirt1	↓ p53 ↑ SDF-1	NE-induced hypertrophy *In vitro* Hypertension model *In vivo*	↓ hypertrophy ↑ bioavailable NO ↓ apoptosis	[60]
In T1DM: ↑ Sirt1, Sirt2, Sirt3, and Sirt5. In T2DM: ↑ Sirt1 and Sirt2 ↓ Sirt3, which was initially elevated	↓ B-MHC ↓ Akt	T1DM-induced cardiomyopathy *In vivo* T2DM-induced cardiomyopathy *In vivo*	In T1DM rats: ↓ cardiac atrophy In T2DM rats: ↓ cardiac hypertrophy	[61]
↑ Sirt1, Sirt3, Sirt4, and Sirt7	↓ caspase 3	H_2_O_2_-induced apoptosis *In vitro*	↓ apoptosis	[62]
Most effects abolished when using sirtinol	↑ SOD1, SOD3, GPx1, catalase. ↓ NOX2, NOX4 ↑ GTP cyclohidrolase 1 and biopterin	*In vivo* Apo-lipoprotein E Knockout mice	↓Oxidative stress Reversed eNOS uncoupling	[63]

AMPK: adenosine monophosphate-activated kinase; Ang II: angiotensin II; B-MHC: myosin heavy chain B; Cyt-c: cytochrome c; Dox: doxorubicin; FS: fractional shortening; Gpx1: glutathione peroxidase 1; GSH: glutathione; LVEF: left ventricular ejection fraction; NE: norepinephrine; NOX: NAD(P)H oxidase; PGC1-*α*: peroxisome proliferator activator of transcription (PPARy) co-activator 1*α*; TAC: transverse aortic constriction; T1DM: type 1 diabetes mellitus; T2DM: type 2 diabetes mellitus; SDF-1: stroma cell derived factor 1; SHD: succinate dehydrogenase; SOD: superoxide dismutase. USP7: ubiquitin-specific-processing protease 7.

**Table 2:** Cardioprotective effect and mechanism of action of curcumin in preclinical studies.

Target HDAC or HAT	Molecular pathway	Experimental model	Cardiovascular effect	Reference
↑ Sirt1	↓ TGF-*β*, Col III, Col I	TAC induced myocardial infraction *In vivo* Ang II-Induced hypertrophy *In vitro*	↓ infract area ↓ fibrosis ↓ hypertrophy	[77]
↑ Sirt1	↑ SOD ↑ Bcl2, ↓ Bax	Isolated ischemia-reperfusion model Ex vivo TAC induced myocardial infraction *In vivo* Simulated ischemia-reperfusion model *In vitro*	Improved post-ischemic cardiac function ↓ myocardial infract size ↓ apoptotic index ↓ oxidative stress Preserved serum CK activity ↓ LDH serum levels	[42]
↑ Sirt1	↑ eNOS ↓ p21	H_2_O_2_-induced endothelial premature senescence *In vitro*	↓ premature senescence ↓ oxidative stress ↓ apoptosis Preverved NO synthesis	[79]
↑ Sirt1	↑ AMPK*α* ↑ LXR-*α* ↑ ATP binding cassette transporter 1	Atherogenic model *In vitro*	Antiatherogenic ↓ cellular cholesterol ↑ cholesterol efflux from THP-1	[78]
↓ p300-HAT	↓ acetylation of histones 3 and 4	LPS-induced cardiac hypertrophy *In vivo*	↓ cardiac hypertrophy	[83]
↓ p300-HAT	↓ TGF-*β*/Smad2	High glucose-induced cardiac hypertrophy *In vitro* Streptozotocin-induced cardiac dysfunction *In vivo*	↓ cardiac hypertrophy ↓ extracellular matrix production ↑ diastolic function	[81]
↓ p300-HAT	↓ GATA4 ↓ NF-*κ*B ↓ acetylation of histones 3 and 4	TAC induced myocardial Infraction *In vivo* PE-induced hypertrophy *In vitro*	↓ LV wall thickness Preserved systolic function ↓ hypertrophy	[77]
↓ p300-HAT	↓ Ac-p53 ↓ ANF, *β*-MHC ↓ Bax, Cyt c, caspase 3, and PARP	TAC induced myocardial Infraction *In vivo* Ang II-Induced hypertrophy *In vitro*	↓ hypertrophy ↓ apoptosis	[80]
↓ p300-HAT	↓ GATA4 ↓ p53	Hypoxia-induced hypertrophy model *In vitro*	Stabilized mitochondrial membrane potential Restored lactate, acetyl-coA pyruvate, and glucose levels	[82]

AMPK: adenosine monophosphate-activated kinase; ANF: atrial natriuretic factor; Ang II: angiotensin II; B-MHC: myosin heavy chain B; CK: creatine kinase; Cyt-c: cytochrome c; EF: ejection fraction; eNOS: endothelial nitrix oxide synthase; LDH: lactate dehydrogenase; LXR-*α*: liver X receptor *α*; LV: left ventricular; NE: norepinephrine; PAI-I: plasminogen activator inhibitor 1; PARP: poly(ADP-ribose) polymerase; PGC1-*α*: peroxisome proliferator activator of transcription (PPARy) coactivator 1*α*; PE: phenylephrine TAC: transverse aortic constriction; SOD: superoxide dismutase.

**Table 3:** Other emerging cardioprotective phytochemicals regulating protein acetylation.

Phytochemical	Target HDAC or HAT	Molecular pathway	Model	Cardiovascular effect	Reference
Honokiol	↑ Sirt3	↓ collagen, B-MHC, and ANF	TAC induced heart failure model *In vivo* PE and Ang II-induced cardiac hypertrophy *In vitro*	Blocks cardiac hypertrophic response Ameliorates preexisting hypertrophy ↓ oxidative stress	[37]
Oroxylin A	↑ Sirt3	↑ aldehyde dehydrogenase	Insulin-induced cardiac dysfunction *In vitro*	Preserved cardiac myocyte contractility	[87]
Epigallocatechin-3-gallate	↑ Sirt1	↑ AMPK-*α* ↑ eNOS	High-fat diet-induced hypercholesterolemia *In vivo*	↓ serum cholesterol ↓ oxidative stress Improved morphology of myocardial tissue	[90]
		↓ Ac-FoxO1 ↓ Nrf2	High-glucose-induced-autophagy *In vitro*	↓ ROS ↓ autophagy	[89]
Quercetin	↑ Sirt1	↑ AMPK-*α* ↑ eNOS ↓ NOX2 ↓ NOX4 ↓ NF-*κ*B	OxLDL-induced endothelial oxidative stress *In vitro*	Preserved mitochondrial function ↓ inflammation	[91]
					
Berberine	↑ Sirt1	↑ SOD ↑ Bcl-2 ↓ Bax, caspase 3	Ischemia/reperfusion-induced myocardial Infraction *In vivo* Simulated ischemia/reperfusion model *In vitro*	↓ infract size ↓ oxidative stress ↓ apoptosis ↓ LDH Maintained LVEF and LVFS Inhibited increase in IL-6 and TNF-*α*	[92]
Bakuchiol	↑ Sirt1	GC-1*α* ↑ Bcl2 ↓ Bax, caspase 3 ↑ SOD, SDH, Cyt-c oxidase	Ischemia reperfusion-induced myocardial infraction Ex vivo Simulated ischemia/reperfusion model *In vitro* Rat cardiac myocytes	↓ apoptosis ↓ oxidative stress Maintained mitochondrial bioenergetics	[93]
n-Tyrosol	↑ Sirt1	↑ Akt ↑ eNOS ↑ Foxo3a	TAC induced myocardial infraction *In vivo*	↓ infract size ↓ apoptosis ↓ fibrosis ↑ LVIDd ↑ EF ↑ FS	[94]
*α*-Lipoic acid	↑ Sirt1	↓ PARP-2	TAC-induced cardiac hypertrophy *In vivo* Ang II-induced hypertrophy *In vitro*	↓ cardiac hypertrophy	[95]
Docosahexaenoic acid	↑ Sirt1	↑ eNOS	*In vitro* Ex vivo	↑ NO synthesis ↑ bioavailable NO	[26]
Sulforaphane	↑ Sirt1	↑ Nrf2, NQo1, HO-1 ↓ PAI-I, TNF-*α*, CTFG, TGF-*β* Preserved LKB1/AMPK/PGC-1*α*	T2DM-induced cardiomyopathy *In vivo*	↓ cardiac remodeling ↓ cardiac dysfunction ↓ cardiac lipid accumulation ↓ oxidative stress ↓ inflammation ↓ fibrosis	[96]
Caffeic acid ethanolamide	↑ Sirt1 ↑ Sirt3	↑ SOD, HIF1-*α*	Isoproterenol-induced cardiac dysfunction *In vivo* *In vitro*	Restored oxygen consumption rates Preserved ATP levels ↓ cardiac remodeling ↓ oxidative stress Preserved mitochondrial function	[97]

AMPK: adenosine monophosphate-activated kinase; ANF: atrial natriuretic factor; Ang II: angiotensin II; B-MHC: myosin heavy chain B; CTFG: connective tissue growth factor; Cyt-c: cytochrome c; Dox: doxorubicin; EF: ejection fraction; eNOS: endothelial nitrix oxide synthase; FS: fractional shortening; HIF1-*α*: hypoxia inducible factor 1-α; HO-1: heme oxygenase; LDH: lactate dehydrogenase; LKB1; liver kinase B 1; LVID internal diameter in diastole; left ventricular, LVEF: left ventricular ejection fraction; NE: norepinephrine; NQo1: NAD(P)H quinone dehydrogenase 1; PAI-I: plasminogen activator inhibitor 1; PARP-2: poly(ADP-ribose) polymerase 2; PGC1-*α*: peroxisome proliferator activator of transcription (PPARy) coactivator 1*α*; PE: phenylephrine TAC: transverse aortic constriction; T2DM: type 2 diabetes mellitus; SHD: succinate dehydrogenase; SOD: superoxide dismutase.
